# Evaluation of the role of remission status in a heterogeneous limited disease small-cell lung cancer patient cohort treated with definitive chemoradiotherapy

**DOI:** 10.1186/s12885-016-2245-x

**Published:** 2016-03-14

**Authors:** Farkhad Manapov, Maximilian Niyazi, Sabine Gerum, Olarn Roengvoraphoj, Chukwuka Eze, Minglun Li, Guido Hildebrandt, Rainer Fietkau, Gunther Klautke, Claus Belka

**Affiliations:** Radiation Oncology, Ludwig-Maximilian University Munich, Marchioninistrasse 15, 81377 Munich, Germany; Radiation Oncology, University of Rostock, Südring 75, 18059 Rostock, Germany; Radiation Oncology, Friedrich-Alexander University Erlangen-Nuernberg, Universitätsstrasse 27, 91054 Erlangen, Germany; Radiation Oncology, Klinikum Chemnitz, Alte Marienberger Strasse 52, 09405 Chemnitz, Germany

**Keywords:** Remission, Chemoradiotherapy, Limited disease, Small-cell, Lung cancer

## Abstract

**Background:**

The role of remission status in limited disease (LD) small-cell lung cancer (SCLC) patients treated with definitive chemoradiotherapy (CRT) remains to be finally clarified.

**Methods:**

Individual data from 184 patients treated with definitive CRT concurrently or sequentially were retrospectively reviewed. Kaplan-Meier analysis as well as univariate and multivariate Cox regression models were used to describe survival within patient subgroups defined by remission status.

**Results:**

71 (39 %) patients were treated in the concurrent, 113 (61 %) in the sequential CRT mode. Prophylactic cranial irradiation (PCI) was applied in 71 (39 %) patients. 37 (20 %) patients developed local, while 89 (48 %) distant recurrence. 58 (32 %) patients developed metachronous brain metastases. Complete, partial remission and non-response (defined as stable and progressive disease) were documented in 65 (35 %), 77 (42 %), and 37 (20 %) patients, respectively. In complete responders median overall survival was 21.8 months (95CI: 18.6 – 25) versus 14.9 (95 % CI: 11.7 – 18.2) (*p* = 0.041, log-rank test) and 11.5 months (95 % CI: 8.9 – 15.0) (*p* < 0.001, log-rank test) in partial and non-responders, respectively. The same effect was documented for the time to progression and distant metastasis-free survival. In the multivariate analysis achievement of complete remission as a variable shows a trend for the prolonged time to progression (*p* = 0.1, HR 1.48) and distant metastasis-free survival (*p* = 0.06, HR 1.63) compared to partial responders and was highly significant compared to non-responders.

**Conclusion:**

In this treated heterogeneous LD SCLC patient cohort complete remission was associated with longer time to progression, distant metastasis-free and overall survival compared to the non- and especially partial responders.

## Background

SCLC accounts for about 13 % of all lung cancer cases with one third of the patients presenting with LD [1]. Due to the early tendency to systemic dissemination, LD SCLC has a relatively rapid course with a median survival for treated patients of approximately one and half a years [1]. Multimodality treatment consisting of chemotherapy and thoracic radiation therapy (TRT) represents a key treatment stone. Additionally, PCI has shown to improve overall survival due to prevention of brain metastasis (BM) [2, 3]. Consecutive meta-analyses for LD SCLC reported better long-term outcome when platinum-based chemotherapy and early concurrent TRT are applied [4, 5]. De Ruysscher et al. found that a short time interval between the first day of any treatment and the last day of TRT is associated with improved overall survival (OS) [6]. Another retrospective study demonstrated that duration of CRT, itself, correlates with OS in LD SCLC patients with poor initial performance status (PS) successfully treated with multimodality therapy [7].

In 2013 Sun et al. published a phase III study investigating the timing of TRT in the course of chemotherapy in LD SCLC [8]. No differences were found in the remission rate and survival between early and late irradiation groups. However, complete response was significantly associated with better OS. A 1997 published trial on the timing of TRT has already described significantly higher complete remission rates associated with better long-term outcome in the early versus late irradiation group [9]. Correlation between remission status after CRT and brain-metastasis free survival in LD SCLC has also been previously documented [10].

The aim of the present study was firstly to establish a correlation between response to multimodality treatment and survival in a heterogeneous LD SCLC patient cohort and secondly to compare different survival parameters in the subgroups of treatment responders, e.g. complete versus partial remission.

## Methods

### Patients

One hundred eighty-four patients from two institutions with initial PS score of WHO 0–3 were diagnosed with LD (UICC Stage I-III) SCLC and successfully treated with definitive CRT in concurrent or sequential modes from 1998 to 2011. Diagnosis was histologically proven in all patients. LD was defined as disease confined to one hemithorax with or without contralateral mediastinal and ipsilateral supraclavicular lymph node involvement, according to Murray et al. [11]. Evidence of pleural effusion and involvement of the contralateral supraclavicular and/or hilar lymph nodes was considered as an exclusion criterion [12]. In all patients initial staging included bronchoscopy with biopsy, CT scans of the chest and abdomen, bone scintigraphy and contrast-enhanced cranial MRI. All patients provided written informed consent before they started treatment. Retrospective study was approved by the University of Munich Ethic Committee.

### Chemoradiotherapy

Concurrent CRT mode was conducted in 71 (39 %) patients and consisted of TRT starting with the first or second cycle of chemotherapy followed by two to four consolidation cycles. The sequential mode of treatment was applied in 113 (61 %) patients consisting of four to six chemotherapy cycles followed by TRT. The most common chemotherapy regimen was a combination of cisplatin either with etoposide or irinotecan. Chemotherapy was given in a 28-day cycle in patients treated with concurrent CRT and in a 21-day cycle in patients treated with sequential CRT according to Takada et al. [13]. TRT was delivered on the linac with megavoltage equipment (8–15 MV) using a coplanar multiple field technique. Three-dimensional CT-simulated treatment planning was performed. Planning target volume was defined as a primary tumour bulk including involved lymph nodes visualised on the pre-therapeutic CT with 1.0 cm margin. 96 % patients were treated 5 days a week with daily fractions of 1.8/2.0 Gy to a total dose of at least 54 Gy (range: 54 – 66Gy). 4 % of patients were treated with hyperfractionated accelerated TRT according to Turrisi AT et al. [14]. After completion of CRT 71 patients (39 %) with good partial and complete remission were treated with PCI (daily 2 Gy to a total dose of 30–36 Gy).

### Response assessment

Response evaluation was done within two weeks after completion of CRT and based on CT scanning of thorax and abdomen as well as bone scintigraphy. Contrast-enhanced cranial MRI was routinely performed before commencing PCI to exclude BM (Brain metastasis). Follow-up care was performed every 3 months during the first two years and every 6 months from the third year onwards. Response evaluation was based on the CT scans and performed by radiologist. Tumor response was defined according to Response Evaluation Criteria in Solid Tumors criteria [15]. Complete remission was defined in cases where staging did not demonstrate any signs of tumor and bronchoscopy revealed a tumor-free biopsy.

### Statistics

All patients were recorded until death. There is no median follow-up due to the fact that the majority of patients died; therefore follow-up was as complete as possible. Survival rates were analysed according to Kaplan-Meier method and were measured from the date of initial diagnosis using SPSS 16.0 software. Kaplan-Meier analyses (pair-wise comparisons) were used to compare survival curves for the complete remission, partial remission and non-response (stable and progressive disease) subgroups. Remission status was also analysed for its association with time to progression (TTP), distant metastasis-free survival (DMFS) and overall survival (OS) by univariate and multivariate Cox regression models after adjustment for other prognostic factors (borderline significant factors in the univariate analysis).

## Results

### Patient and treatment characteristics

Patient characteristics are described in Table 1. Of 184 patients treated, 111 (60 %) were men and 73 (40 %) were women. Median age at diagnosis was 63 years (range: 34–83). 34 (19 %) patients were older than 70 years. Median PS according to WHO for the entire cohort was 1 (range: 0 to 3). 71 (39 %) patients were treated with concurrent and 113 (61 %) sequential treatment modes. T3/4-Stage disease was diagnosed in 101 (55 %) patients. 110 (60 %) patients presented with N-Stage 2 or 3. T1-T2 (<5 cm) primary tumors without lymph node involvement were only detected in five (3 %) patients. Sufficient data on T- and N-stage were missing in 26 (14 %) and 35 (19 %) cases, respectively. There were no significant differences in regard to age, sex, PS and TNM-stage between patients treated in the concurrent and sequential groups. Platinum-based chemotherapy was applied in 164 (89 %) patients. 36 (20 %) patients were treated with less than four cycles of chemotherapy. PCI was applied in 71 (39 %) patients with good partial or complete remission. Median duration of multimodality treatment was 165 (range: 16–327) days. Median duration of TRT was 43 (range: 16–76) and of chemotherapy 108 (range: 5–233) days, respectively.

**Table 1:** Patient- and treatment characteristics

Characteristics	Number of Patients (*N* = 184)	%
Age at diagnosis		
Median 63 (range 34–83)		
>70 years	34	19
Sex		
M	111	60
F	73	40
CRT mode		
Sequential	113	61
Concurrent	71	39
Chemotherapy		
Platinum based	164	89
Non platinum based	20	11
Chemotherapy Cycles		
> = 4	148	80
<4	36	20
PCI		
yes no	71 113	39 61

### Treatment response

Treatment response to definitive CRT is described in Table 2. Objective response was found in 142 (77 %) patients. Complete remission was documented in 65 (35 %) patients and was confirmed with bronchoscopy. 77 (42 %) patients had a partial remission. 37 (20 %) patients had non-response (stable or progressive disease). A lack of data on remission status was documented in 5 (3 %) cases. Local recurrence was found in 37 (20 %) patients. 89 (48 %) patients developed distant metastases. Metachronous BMs were detected in 58 (32 %) patients. Median OS, DMFS and TTP for the entire cohort were 16.8 (95 CI: 14.8 – 18.9), 18.2 (95 CI: 14.1 – 22.2) and 14.5 months (95 CI: 11.9 – 17.1), respectively. No difference in survival parameters could be found in patients treated with the concurrent versus sequential modes.

**Table 2:** Distribution of treatment response to definitive chemoradiotherapy

Treatment Response	Number of Patients (*N* = 184)	%
Complete remission	65	35
Partial remission	77	42
Non-Response (stable/progressive disease)	37	20
Not validated	5	3
Metachronous brain failure	58	32
Distant failure	89	48
Local failure	37	20

### Remission status and survival

**Fig. 1:**
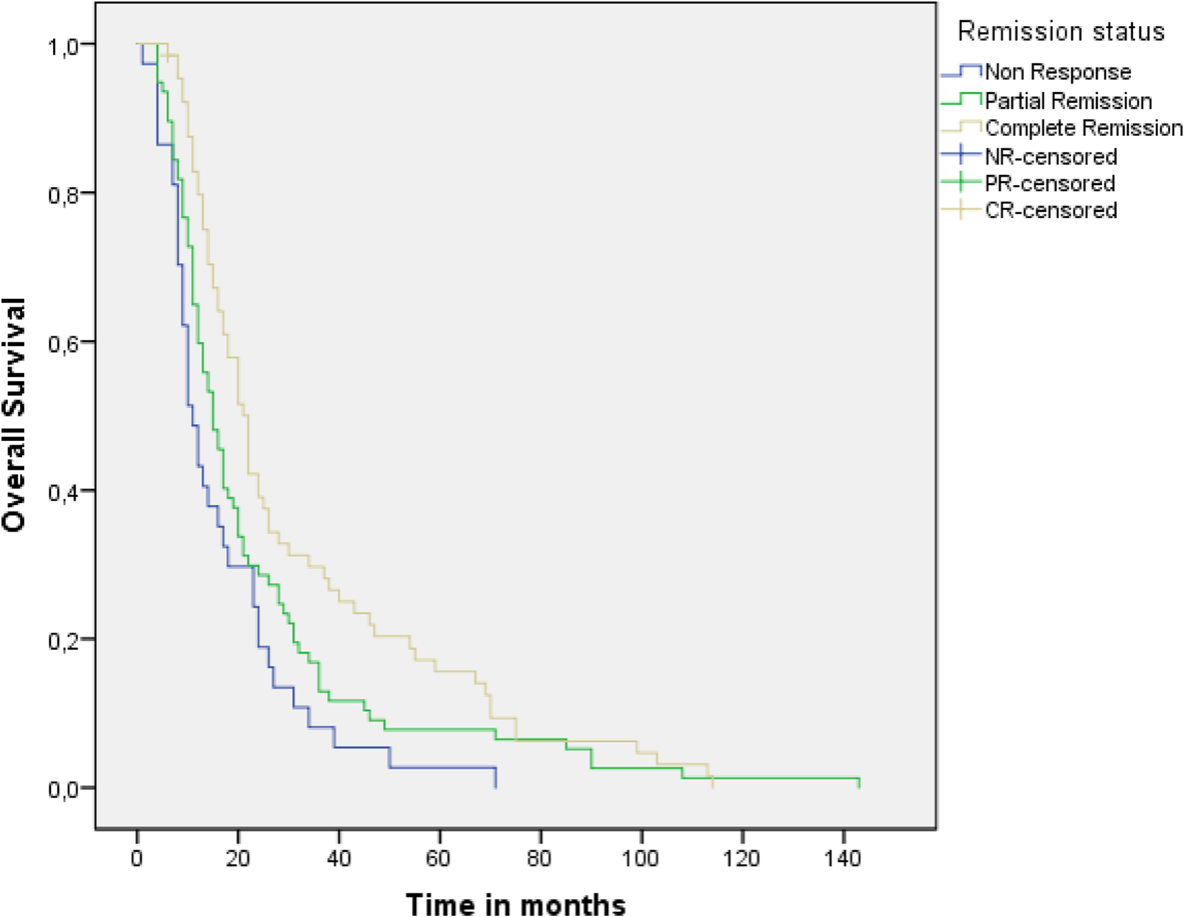
Overall survival in patient subgroups defined by remission status after CRT

**Fig. 2:**
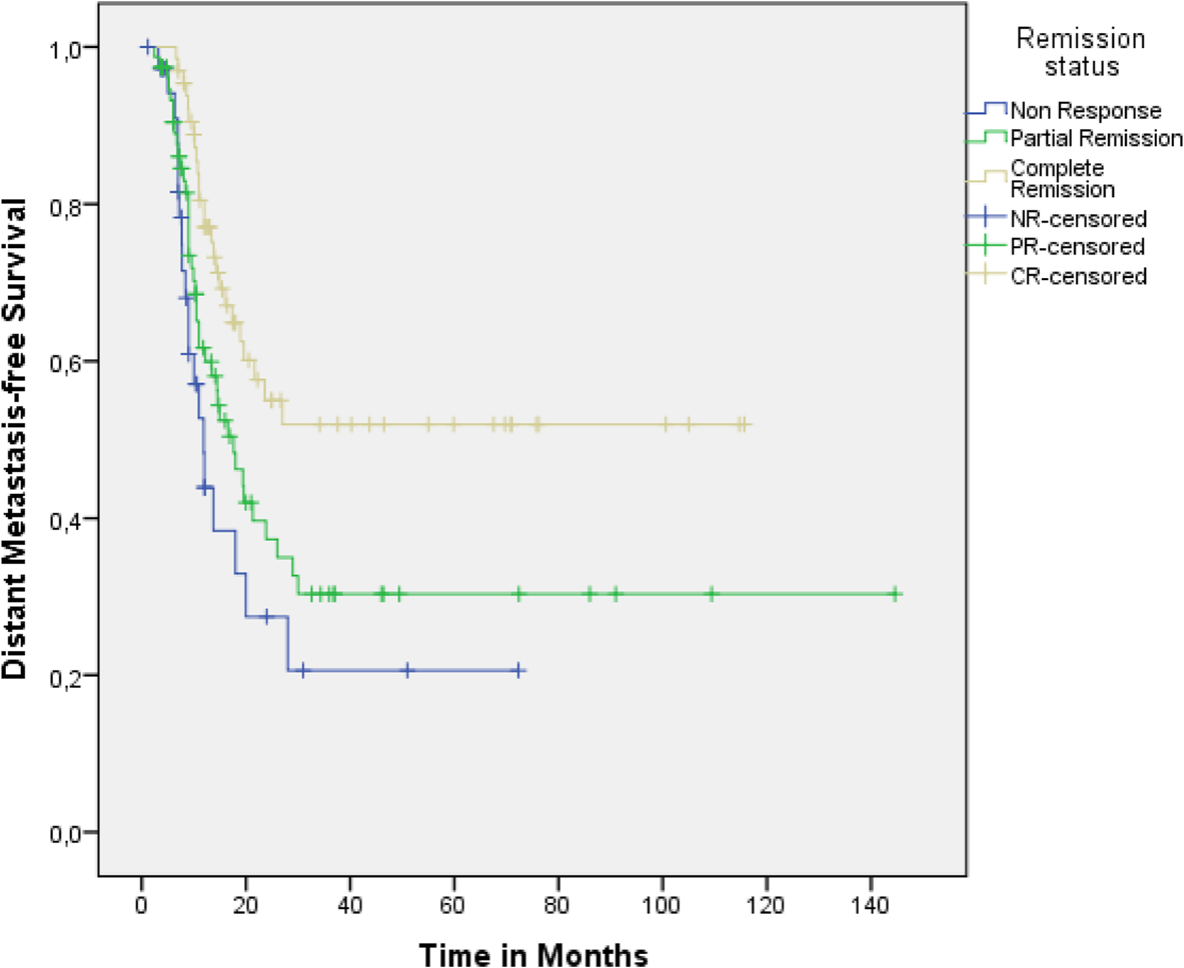
Distant metastasis-free survival in patient subgroups defined by remission status after CRT

**Fig. 3:**
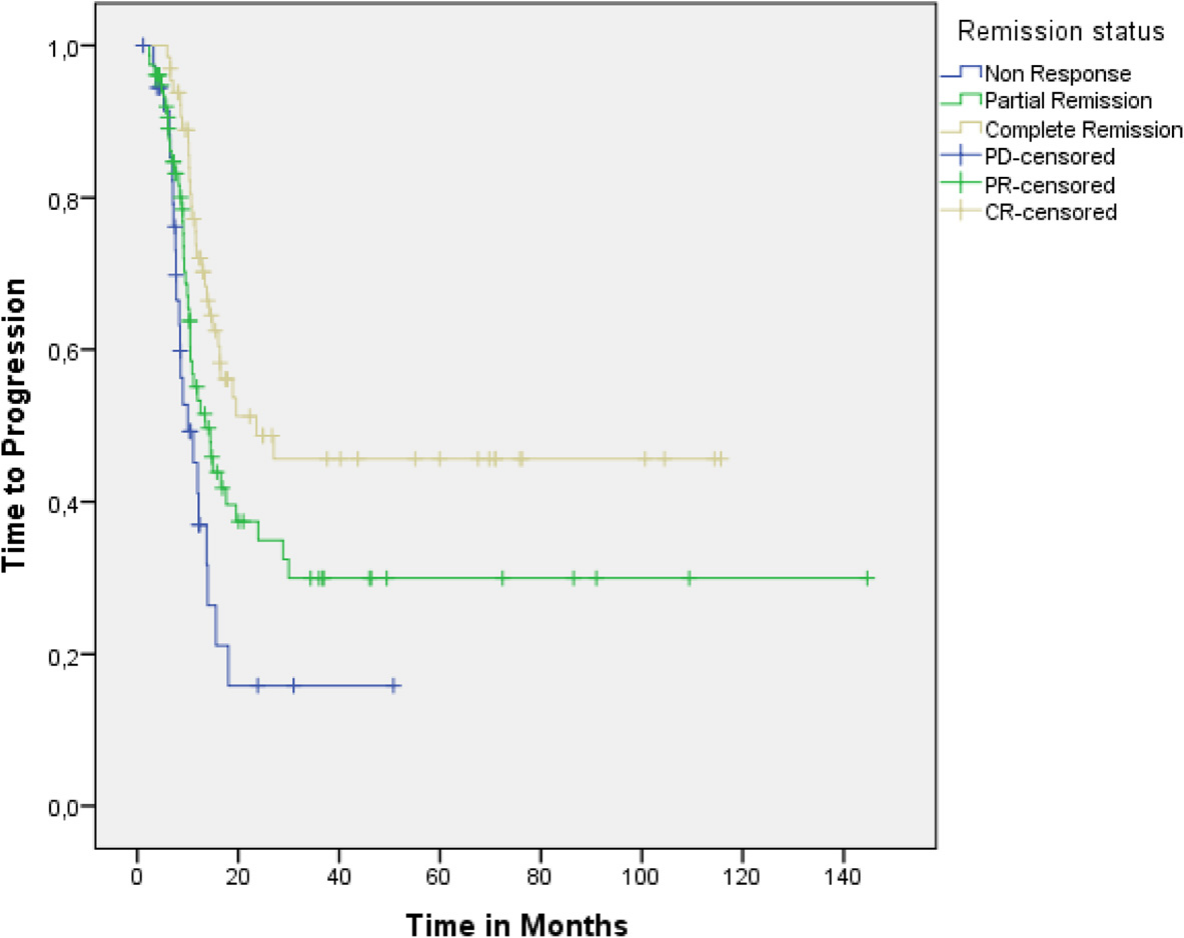
Time to progression in patient subgroups defined by remission status after CRT

Pair-wise comparisons for OS, DMFS and TTP within the patient subgroups defined by remission status were performed. Median OS in complete responders was 21.8 (95 % CI: 18.6 – 25) versus 14.9 (95 % CI: 11.7 – 18.2) (*p* = 0.041, log-rank test) and 11.5 months (95 % CI: 8.9 – 15) (p < 0.001, log-rank test) in partial and non-responders, respectively (Fig. 1). Considering the control of systemic disease, median DMFS in patients with complete remission was not reached (Fig. 2: see Plateau was over 50 %) whereas in partial and non-responders, it was only 16.6 (95 % CI: 11.9 – 21.2) (*p* = 0.009, log-rank test) and 11.9 (95 % CI: 8.9 – 15) (*p* = 0.001, log-rank test) months, respectively. The same effect was also shown for the TTP: in complete responders it was 23.6 versus 13.5 (range: 9.2 – 17.7) (*p* = 0.027, log-rank test) and 10 (range: 6.1 – 13.9) (p < 0.0001, log-rank test) months in patients with partial remission and stable/progressive disease, respectively (Fig. 3: see Plateau).

In the multivariate analysis, comparing survival in complete and partial responders, the trend for prolonged TTP (*p* = 0.1, HR 1.48) and DMFS (*p* = 0.06, HR 1.63) was demonstrated (Table 3). Significantly longer OS, DMFS and TTP in complete responders compared to non-responders were confirmed.

**Table 3:** Survival parameters in the multivariate analysis after adjustment for other prognostic factors

Survival	Complete versus partial remission (HR and *p* value)	Complete remission versus non-response (HR and *p* value)
Median OS	1.267 (95CI: 0.899 – 1.787) *p* = 0.177	2.135 (95CI: 1.392 – 3.275) *p* = 0.001
Median DMFS	1.632 (95CI: 0.978 – 2.724) *p* = 0.061	3.276 (95CI: 1.771 –6.057) *p* < 0.001
Median TTP	1.486 (95CI: 0.912 – 2.422) *p* = 0.1	3.144 (95CI: 1.776 –5.595) *p* < 0.0001

## Discussion

The aim of this retrospective analysis was to establish the role of remission status in LD SCLC patients treated with chemotherapy and TRT without surgery and to compare survival parameters in the different subgroups defined by remission status. This study demonstrates a clear correlation between achieved remission after primary multimodality treatment and systemic disease control as well as overall survival. Especially our results show that complete response following CRT was associated with prolonged TTP, DMFS and OS when compared to partial remission.

Disease control becomes of prime importance in the treated LD SCLC due to the early onset of metastases. A number of studies have reported that the absolute majority of patients with LD SCLC will develop a recurrence [1, 12, 16, 17]. Our analysis on the timing of treatment failure in LD SCLC has demonstrated that in more than half of the patients with distant relapse, failure occurred in the first year from initial diagnosis [18]. Hence previous clinical trials have addressed the question of the correlation between treatment response, disease control and outcome after CRT. A phase III trial published in 1997 by Jeremic et al. firstly showed higher complete remission rates in patients treated with early compared to late concurrent CRT correlated with better long-term survival [9]. However, remission status itself was not analyzed as a prognostic factor. Sixteen years later Sun et al. conducted a phase III study on the timing of TRT concurrent with chemotherapy with complete remission rate as the primary endpoint. Early and late TRT arms were found to be identical concerning remission status and survival rates. The trial demonstrated that complete responders independent of the timing of TRT have significantly better prognosis compared to the rest of the treated patients [8]. In contrast to the above mentioned studies, the present analysis was conducted in a heterogeneous patient cohort and aimed to compare survival parameters between complete, partial and non-responders. The importance of the achievement of complete remission for the TTP, DMFS and OS was emphasized. This fact may be considered in the planning and assessment of future multimodality trials for LD SCLC.

The relevance of tumor shrinkage or downstaging during the course of CRT was already investigated in several smaller studies [19–21]. A correlation between early metabolic (before start of TRT) and CT changes of the tumor volume and survival in LD SCLC was described by van Loon et al. [19]. Go et al. revealed that downstaging during CRT can be considered as an independent prognostic factor [20]. Also Fujii et al. reported that the achievement of remission after the first cycle of chemotherapy applied simultaneously with TRT was associated with significantly better 2-year survival rate [21].

A major limitation of the present study is its retrospective nature. However, described treatment response rates and survival correlated well with reported historical data. Another important critical point is the heterogeneity of the analyzed cohort but only 3 % of patients presented with primary tumors <5 cm without lymph-node involvement (UICC Stage I). Comprehensive retrospective acquisition of the treatment toxicity was not possible and we decided to analyze only medical charts of the patients who completed definitive CRT without interruptions. Moreover an integration of the PET-CT (Positron emission tomography–computed tomography) could not be exactly evaluated, because fewer than 20 % percent of patients received PET-CTs at initial staging. Nevertheless, present results point out a clinical relevance of the complete remission after definitive CRT and suggest that remission status may be considered as an additional factor in the planning and assessment of multimodality clinical trials for LD SCLC.

## Conclusion

In our retrospective analysis of heterogeneous LD SCLC patient cohort, achievement of complete remission after definitive CRT was associated with a relevant survival advantage compared to the patients with stable/progressive disease and especially partial responders.

## Abbreviations

BM: brain metastasis
CRT: chemoradiotherapy
CT: computed tomography
DMFS: distant metastasis-free survival
LD: limited disease
OS: overall survival
PCI: prophylactic cranial irradiation
PET-CT: Positron emission tomography–computed tomography
PS: performance status
SCLC: small-cell lung cancer
TRT: thoracic radiation therapy
TTP: time to progression
